# Time from first seen in specialist care to surgery does not influence survival outcome in patients with upfront resected pancreatic adenocarcinoma

**DOI:** 10.1186/s12893-021-01409-7

**Published:** 2021-12-07

**Authors:** M. Brugel, O. Bouché, R. Kianmanesh, L. Teuma, A. Tashkandi, J. M. Regimbeau, P. Pessaux, B. Royer, R. Rhaiem, C. Perrenot, C. Neuzillet, T. Piardi, S. Deguelte

**Affiliations:** 1 Department of Ambulatory Oncology Care Unit, Centre Hospitalier Universitaire de Reims, Rue du general Koenig, Reims, France; 2 University Reims Champagne-Ardenne (URCA), Reims, France; 3 Digestive and Endocrine Surgery Department, Centre Hospitalier Universitaire de Reims, Rue du général Koenig, Reims, France; 4 Digestive Surgery Department, CHU Amiens Picardie, 1 rond-point du Professeur Christian Cabrol, Amiens, France; 5 University of Picardie Jules-Vernes, 51 boulevard de Chateaudun, Amiens, France; 6 General, Digestive, and Endocrine Surgery, Nouvel Hôpital Civil, 1 quai Louis Pasteur, Strasbourg, France; 7 Université de Strasbourg, Strasbourg, France; 8 General Surgeon, Clinique de Courlancy, 38bis rue de Courlancy, Bezannes, France; 9 Medical Oncology Department, Institut Curie, 35 rue Dailly, Saint-Cloud, France; 10 Versailles Saint-Quentin University, Paris Saclay University, Saint-Cloud, France

**Keywords:** Pancreatic adenocarcinoma, Resectable, Delay, Time to surgery

## Abstract

**Background:**

This study evaluated the impact of time to surgery (TTS) on overall survival (OS), disease free survival (DFS) and postoperative complication rate in patients with upfront resected pancreatic adenocarcinoma (PA).

**Methods:**

We retrospectively included patients who underwent upfront surgery for PA between January 1, 2004 and December 31, 2014 from four French centers. TTS was defined as the number of days between the date of the first consultation in specialist care and the date of surgery. DFS for a 14-day TTS was the primary endpoint. We also analyzed survival depending on different delay cut-offs (7, 14, 28, 60 and 75 days).

**Results:**

A total of 168 patients were included. 59 patients (35%) underwent an upfront surgery within 14 days. Patients in the higher delay group (> 14 days) had significantly more vein resections and endoscopic biliary drainage. Adjusted OS (p = 0.44), DFS (p = 0.99), fistulas (p = 0.41), hemorrhage (p = 0.59) and severe post-operative complications (p = 0.82) were not different according to TTS (> 14 days). Other delay cut-offs had no impact on OS or DFS.

**Discussion:**

TTS seems to have no impact on OS, DFS and 90-day postoperative morbidity.

## Introduction

Pancreatic adenocarcinoma (PA) is one of the most aggressive digestive cancers. Five-year overall survival (OS) rate is estimated below 8% (all stages combined) [1]. This malignancy is expected to be the second leading cause of cancer-related death in Europe by 2030[2].

French actual standard of care for resectable PA is upfront carcinologic surgery followed by adjuvant chemotherapy [3]. Unfortunately, prognosis remains poor despite improvements in surgical technique, perioperative care, diagnosis accuracy, patient selection and more active chemotherapy regimen [4]. New perspectives are needed to increase both survival rates and quality of life for patients diagnosed with PA.

One of the objectives studied in other cancers has been to reduce time to treatment by improving the organization of the care pathway. Time to surgery (TTS) has turned out to be a major prognostic factor associated with survival in several malignancies [5–8]. To date, the impact of TTS on OS and disease-free survival (DFS) in patients diagnosed with resectable PA remains unclear [9].

Moreover, pancreatic resection is one of the most challenging surgery, with significant postoperative morbidity and mortality [10]. Improving preoperative status—by means of biliary drainage, hemostasis correction, prehabilitation with nutritional and adapted physical activity interventions [11]—is needed before surgery as complications are more frequent in unfit patients [12]. Shorter TTS may not allow to optimize prehabilitation but could improve carcinologic prognosis.

This study evaluated the impact of TTS on OS, DFS and postoperative complication rate in patients who underwent upfront curative intent surgical resection of a PA.

## Materials and methods

We retrospectively included patients with upfront resected PA between January 1, 2004 and December 31, 2014 in three tertiary French centers (Reims University Hospital, Amiens University Hospital, Strasbourg University Hospital) and in one private center (Reims Courlancy Clinic). Patients were screened with administrative coding and multidisciplinary tumor board meetings data (MTBM). All cases have been discussed in MTBM, including a senior radiologist and a pancreatic surgeon, as now recommended by the consensus of the International Study Group of Pancreatic Surgery [13]. No systematic preoperative imaging review was made at inclusion.

All patients who underwent upfront curative intent resection for a PA were included. Patients were excluded in case of neoadjuvant treatment or incomplete surgical resection (R2).

Basic baseline clinical, biological (i.e. ECOG performance status (PS), initial symptoms, tumor location, preoperative biliary drainage, bilirubin levels and neutrophil–lymphocyte ratio), pathological and surgical data [i.e. TNM staging (5th, 6th or 7th edition, according to the standard of care at the time of treatment], lymph node ratio (number of invaded lymph nodes/total number of resected lymph nodes), resection margins [R0 or R1 (< 1 mm)], venous and adjacent organ involvement (gastric, colon or left adrenal resection), were collected using medical records. Follow up characteristics such as postoperative complications, adjuvant treatment regimen and evolution of the disease (tumor recurrence, site of recurrence and death) were also collected.

Time to decision (TTD) was defined as the delay in days between first specialized medical interview (gastroenterologist, pancreatic surgeon, or medical oncologist) and MTBM decision. Time to surgery (TTS) was defined as the delay between the first specialized medical interview (gastroenterologist, pancreatic surgeon, medical oncologist) and surgery. The 90-day postoperative complications were assessed using Dindo-Clavien classification and ISGPS definitions [14]. Grade III or higher grade complications were considered as severe [14]. Grade V complications correspond to postoperative death. Follow-up was standard and left to the physician’s discretion according to guidelines prevailing at the time of the treatment [3]. The primary objective was to determine the impact of a shorter (≤ 14 days) TTS in DFS improvement. This threshold was chosen in accordance with literature review and the investigators’ experience [15–20]. Secondary objectives were to evaluate OS and DFS according to other TTS and TTD thresholds (7-, 14-, 28-, 60- and 75-day delay). Ninety-day morbidity rate was compared using a 14-day delay. Endpoint date was set to provide at least a 12-month follow-up (December 31, 2015).

### Statistical analysis

Continuous variables were described as median and interquartile ranges. Categorical variables were described as frequencies expressed with percentages. Groups with different management delays were compared using Mann–Whitney test (non-normal continuous variables) and Chi square test or Fisher test (categorical variables), depending on variable type and sample size. OS was calculated from date of surgery procedure to date of death or censoring at the date of last visit. DFS was calculated from the date of the surgical procedure to the date of progression, death or censoring at the date of last visit [9]. Survival curves were established using the Kaplan–Meier method and compared using Cox proportional-hazards model for univariate and multivariate analyses. We pre-planned several survival analyses stratified on different delay cut-offs (7, 14, 28, 60 and 75 days). Complication rates were analyzed according to a 14-day delay using Mann–Whitney test and logistic regression adjusted for factors with a p < 0.2 in stepwise regression. All data analyses were performed using R (R Development Core Team, 2005). Statistical significance was defined as a p value < 0.05 for all tests.

### Ethics

Patient’s records were anonymized prior to analysis. Database was constituted in accordance with the reference methodology MR004 of the National Commission of Liberties and Informatics. (no. 2206749, 13/09/2018). As per French regulations, no additional ethical review was required.

## Results

### Population characteristics at baseline

**Fig. 1:**
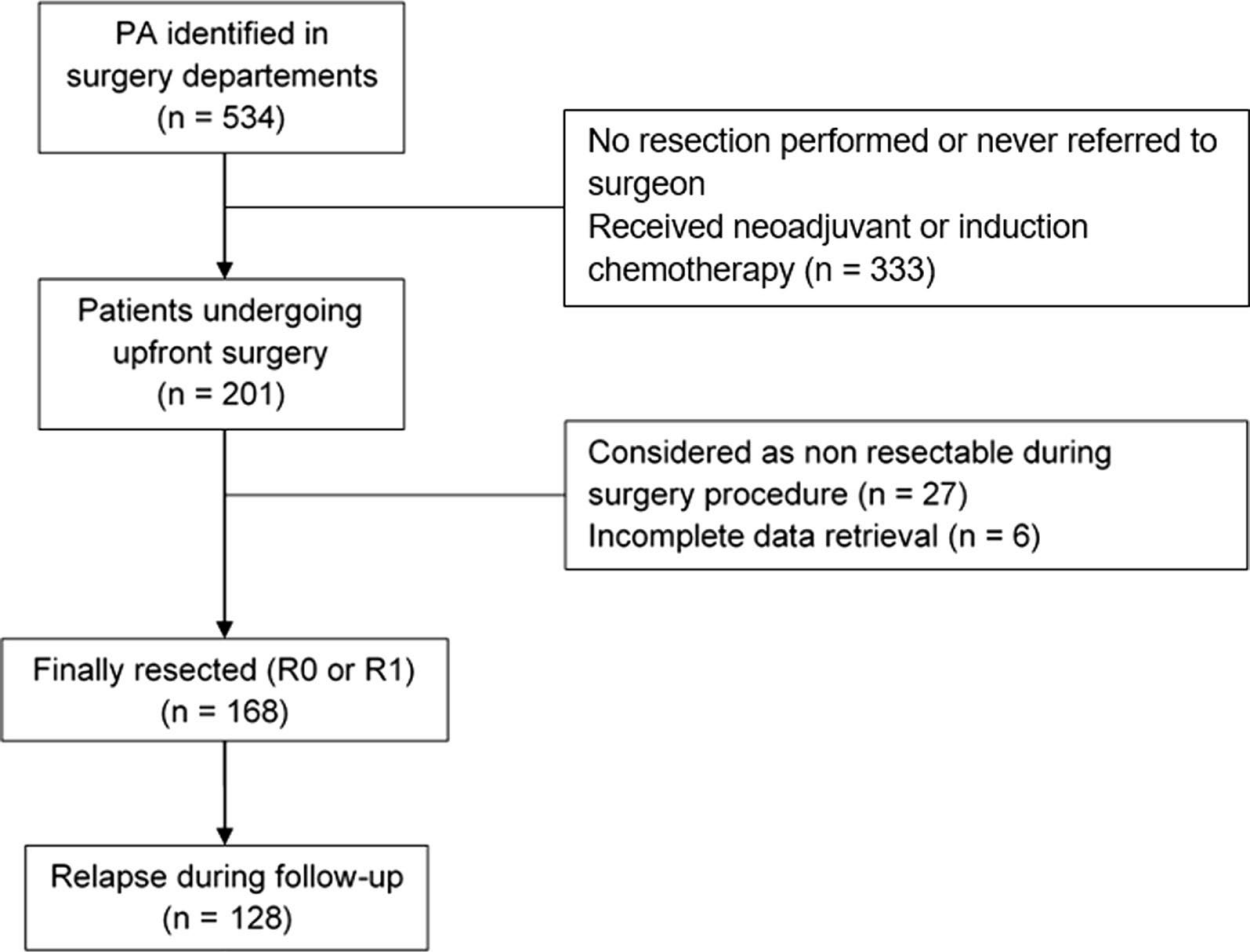
Flowchart. *n* total population; *R* resection status

A total of 534 patients with a PA were screened. Among them, 201 patients underwent an upfront surgery procedure with curative intent; 33 were finally excluded due to unresectable tumor upon surgical reassessment [21] or insufficient data collection [6] yielding a total of 168 included patients. The study flowchart is presented in Fig. 1 (Flowchart).

Patient characteristics are presented in Table 1. Most patients were men (57.7%), with a median age of 66 years (IQR 58–71). ECOG PS was available in 60.2% of the cases. Only four patients were ECOG PS > 1. Jaundice was the main symptom (69%) at first patient visit, and diagnosis was made during a hospital stay in 44.6% of the cases. Tumors were mainly located in the head of the pancreas (79.8%), warranting a biliary drainage in 64 patients (38.1%). Nine of the patients underwent isolated metastases (liver and/or distant lymph nodes) resection, in curative intent at the time of surgery. Median total bilirubin prior to any biliary drainage was 197.5 µmol/L (IQR 135.5–336.75).

**Table 1:** Study population characteristics

A. Baseline characteristics	
Characteristics	n = 168
Inclusion center (%)	
Reims University Hospital	70 (41.7)
Reims Courlancy Clinic	12 (7.1)
Amiens University Hospital	60 (35.7)
Strasbourg University Hospital	26 (15.5)
Male (%)	71 (57.7)
Age, years (median [IQR])	66 [58.00, 71.00]
BMI, kg/m^2^ (median [IQR])	25.33 [22.49, 28.58]
Performance status (%)	
0	70 (41.7)
1	27 (16.1)
2	4 (2.4)
NA	67 (39.9)
Clinical symptoms at presentation (%)	
Jaundice	116 (69.0)
Abdominal pain	53 (31.5)
Weight loss	5 (3.0)
Incidentaloma	6 (3.6)
Other	26 (15.5)
NA	1 (0.6)
Diagnosed during hospital stay (%)	75 (44.6)
Imaging technique used for diagnosis (%)	
Computed tomography	85 (50.6)
Echography	18 (10.7)
Echoendoscopy	37 (22.0)
MRI	21 (12.5)
Other	5 (3.0)
NA	2 (1.2)
Tumor location (%)	
Head	134 (79.8)
Body	13 (7.7)
Tail	18 (10.7)
NA	3 (1.8)
Lymphadenopathy at imaging (%)	27 (16.1)
Endoscopic biliary drainage (%)	58 (34.5)
Radiological biliary drainage (%)	6 (3.6)
Total bilirubin (µmol/L) (median [IQR])	54.00 [9.47, 185.25]
Conjugated bilirubin (µmol/L) (median [IQR])	46.00 [7.20, 148.50]
Neutrophils to lymphocytes ratio (median [IQR])^†^	2.67 [2.00, 3.82]
Time to decision (days) (median [IQR])	9.50 [1.00, 30.50]
Time to surgery (days) (median [IQR])	19.00 [12.00, 36.00]

B. Surgery procedure, histopathological, and follow up characteristics	
Characteristics	n = 168
Type of resection (%)	
Pancreaticoduodenectomy	131 (78.0)
Splenopancreatectomy	28 (16.7)
Left pancreatectomy	4 (2.4)
Total pancreatectomy	5 (3.0)
Vein resection (%)	69 (41.1)
Organ resection (%)^a,b^	10 (6.0)
T (%)	
1	3 (1.8)
2	25 (14.9)
3	128 (76.2)
4	9 (5.4)
x	1 (0.6)
NA	2 (1.2)
N (%)^b^	
0	37 (22.0)
1	129 (76.8)
x	1 (0.6)
NA	1 (0.6)
M (%)^a,b^	
0	157 (93.5)
1	9 (5.4)
x	2 (1.2)
Number of lymphatic nodes sampled (median [IQR])	19.00 [12.00, 25.00]
Number of invaded lymph nodes (median [IQR])^b^	2.00 [1.00, 4.00]
Invaded/sampled lymph nodes (median [IQR])	0.08 [0.00, 0.20]
Resection status (%)^b^	
0	105 (62.5)
1	62 (36.9)
NA	1 (0.6)
Microscopic vascular invasion (%)	
Yes	103 (61.3)
No	25 (14.9)
NA	40 (23.8)
Perineural invasion (%)	
Yes	137 (81.5)
No	10 (6.0)
NA	21 (12.5)
Length of stay (days) (median [IQR])	19.00 [14.00, 27.50]
Postoperative complications (%)	
Yes	109 (64.9)
No	50 (29.8)
NA	9 (5.4)
Hemorrhage (%)^a,b^	
Yes	22 (13.1)
No	87 (51.8)
NA	59 (35.1)
Fistula (%)^a,b^	
Yes	42 (25.0)
Fistula grade (%)	
1 A	22 (52.4)
2 B	13 (31)
3 C	6 (14.3)
NA	1 (2.4)
No	66 (39.3)
NA	60 (35.7)
Dindo-Clavien classification (%)	
Benign	124 (73.8)
0	63 (37.5)
1	18 (10.7)
2	43 (25.6)
Severe^a,b^	41 (24.4)
3a	11 (6.5)
3b	13 (7.7)
4a	5 (3.0)
4b	2 (1.2)
5	10 (6.0)
NA	3 (1.8)
Follow-up (days) (median [IQR])	651.5 [374, 1077.2]
Adjuvant chemotherapy (%)	141 (83.9)
Number of chemotherapy cycles (median [IQR])	6.00 [5.50, 6.00]
Adjuvant radiotherapy (%)	13 (7.7)
Disease recurrence (%)	128 (76.2)
Local disease recurrence (%)	6 (3.6)
Lymph node recurrence (%)	37 (40.7)
Liver recurrence (%)^a^	45 (49.5)
Peritoneal recurrence (%)	15 (16.5)
Other (%)	25 (27.5)
Disease-free survival (days) (median [IQR])	363.00 [214.75, 534.25]
Death (%)	114 (67.9)

*n* total population; *IQR* interquartile range; *kg/m*^2^ kilograms per square meter; *BMI* body mass index; *NA* not available; *MRI* magnetic resonance imaging

^a^Variable associated with overall survival using univariate Cox proportional-hazards model

^b^Variable associated with disease free survival using univariate Cox proportional-hazards model

### Treatment characteristics

Pancreaticoduodenectomy was performed in 131 patients, (78%). Mesenteric superior vein was resected in 69 patients (41.1%). Other organs were resected in only 10 patients (6%) (Table 1B). Median length of hospital stay was 19 days (IQR 14–27.5). TNM stage on the final pathology report was mainly T3 (76.2%) and N1 (76.8%). A median of two lymph nodes were positive at pathological analysis (IQR 1–4) for a lymph node ratio calculated at 0.08 (IQR 0.00–0.2). Postoperative complication data were available in 159 patients (94.6%). Eighty-seven (51.8%) and 66 patients (39.3%) experienced hemorrhage and fistulas, respectively. Post-operative complications were considered severe in 41 patients (24.4%). Ten patients (6%) died of post-operative complications (Dindo-Clavien class 5).

Adjuvant chemotherapy was administered in 141 patients (83.9%), mainly gemcitabine. A median of 6(IQR 5.5–6) chemotherapy cycles were administered. was Adjuvant radiotherapy was used for 13 patients (7.7%).

Median follow-up lasted 651.5 days (IQR 374, 1077.2), approximatively 21.5 months. Recurrence was identified for 128 patients (76.2%), and classified as at least distant/metastatic (95.3%, 122 patients), or only locoregional (4.7%, 6 patients). A total of 114 (67.9%) patients had died at study endpoint date.

### Delay analysis

Median TTD was 9.5 days (IQR 1–30.5). Median TTS was 19 days (IQR 12–36).

Patient characteristics stratified upon TTS inferior to 14 days are presented in Table 2. A total of 59 patients (35%) had their tumor resected less than 14 days after first consultation. Patients in the longer delay group had more frequent vein resections, endoscopic biliary drainage (p < 0.001), jaundice (p = 0.044), and higher median total serum bilirubin levels (p < 0.001).

**Table 2:** Study population characteristics stratified by a fourteen-day-time-to-surgery

A. Baseline characteristics			
Characteristics (n = 168)	≤ 14 days	> 14 days	*p*
n	59	106	
Male (%)	38 (64.4)	58 (54.7)	0.334
Age (median [IQR])	65.00 [57.00, 69.50]	67 [59, 73]	0.109
BMI (median [IQR])	25.52 [23.20, 29.49]	25.10 [22.50, 28.33]	0.474
Performance status (%)			0.747
0	23 (65.7)	47 (71.2)	
1	10 (28.6)	17 (25.8)	
2	2 (5.7)	2 (3.0)	
Jaundice (%)	47 (79.7)	67 (63.2)	0.044*
Diagnosed during hospital stay (%)	30 (50.8)	45 (42.5)	0.382
Tumor location (%)			0.3
Head	51 (87.9)	82 (78.1)	
Body	3 (5.2)	10 (9.5)	
Tail	4 (6.9)	13 (12.4)	
Lymphadenopathy at imaging (%)	9 (21.4)	18 (20.2)	1
Endoscopic biliary drainage (%)	10 (20.8)	46 (65.7)	< 0.001*
Radiological biliary drainage (%)	1 (2.1)	5 (7.1)	0.422
Total bilirubin (µmol/L) (median [IQR])	173.00 [74.25, 304.50]	21.00 [8.00, 84.75]	< 0.001*
Conjugated bilirubin (µmol/L) (median [IQR])	140.50 [60.50, 236.25]	22.00 [4.00, 76.50]	< 0.001*
Neutrophil-to-lymphocyte ratio (median [IQR])	2.67 [2.06, 4.09]	2.68 [1.92, 3.52]	0.223

B. Surgery procedure, histopathological, and follow up characteristics stratified on a 14 days-time to surgery *n* total population; *IQR* interquartile range; *NA* not available; *statistical significance			
Characteristics (n = 168)	≤ 14 days	> 14 days	*p*
n	59	106	
Type of resection (%)			0.497
Duodenopancreatectomy	50 (84.7)	79 (74.5)	
Splenopancreatectomy	7 (11.9)	20 (18.9)	
Left pancreatectomy	1 (1.7)	3 (2.8)	
Total pancreatectomy	1 (1.7)	4 (3.8)	
Vein resection (%)	16 (27.1)	52 (49.1)	0.01*
Organ resection (%)	4 (6.8)	6 (5.7)	1
T (%)			0.375
1	0 (0.0)	3 (2.9)	
2	6 (10.2)	18 (17.3)	
3	49 (83.1)	77 (74.0)	
4	4 (6.8)	5 (4.8)	
x	0 (0.0)	1 (1.0)	
N (%)			0.488
0	11 (18.6)	26 (24.8)	
1	48 (81.4)	78 (74.3)	
x	0 (0.0)	1 (1.0)	
M (%)			0.111
0	53 (89.8)	101 (96.2)	
1	6 (10.2)	3 (2.9)	
x	0 (0.0)	1 (1.0)	
Invaded/sampled lymph nodes (median [IQR])	0.06 [0.00, 0.15]	0.10 [0.00, 0.20]	0.403
Resection status = 1 (%)	23 (39.0)	38 (36.2)	0.852
Microscopic vascular thrombosis (%)	38 (84.4)	64 (79.0)	0.612
Perineural invasion (%)	50 (92.6)	86 (93.5)	1
Hemorrhage (%)	10 (25.6)	11 (16.2)	0.351
Fistula (%)	12 (31.6)	28 (41.2)	0.442
Severe complications (%)	15 (25.4)	26 (24.5)	1
Length of stay (days) (median [IQR])	21.00 [15.00, 28.00]	19.00 [13.25, 25.00]	0.416
Adjuvant chemotherapy (%)	52 (91.2)	87 (82.9)	0.222
Number of chemotherapy cycles (median [IQR])	6.00 [5.50, 6.00]	6.00 [5.33, 6.00]	0.710
Adjuvant radiotherapy (%)	4 (7.0)	9 (8.6)	1.000
Local disease recurrence (%)	0 (0)	6 (5.7)	
Distant disease recurrence (%)	48 (81.3)	72 (74.5)	0.761
Lymph node recurrence (%)	12 (35.3)	24 (43.6)	0.578
Liver recurrence (%)	21 (61.8)	24 (43.6)	0.149
Peritoneal recurrence (%)	5 (14.7)	10 (18.2)	0.893
Other (%)	10 (29.4)	14 (25.5)	0.871
Death (%)	41 (69.5)	70 (66.0)	0.779
Time from surgery to adjuvant chemotherapy (days) (median [IQR])	55.00 [43.50, 74.50]	54.00 [42.75, 63.00]	0.543

*n* total population; *IQR* interquartile range; *NA* not available

*Statistical significance according to Mann–Whitney test (nonnormal continuous variables) or Chi-square test (categorical variable)

**Fig. 2:**
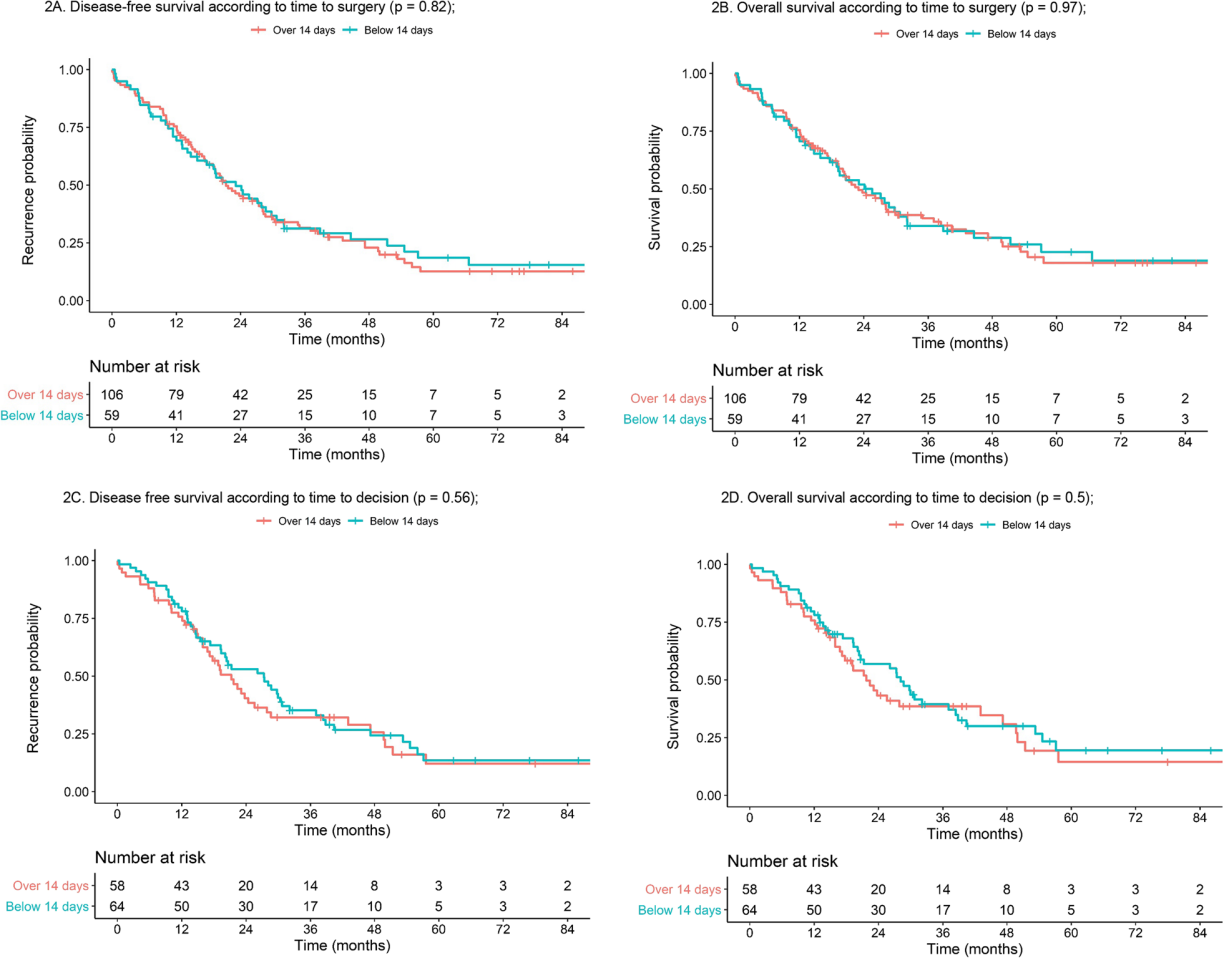
Disease-free and overall survival according to time to surgery and time to decision. **A** Disease-free survival according to time to surgery (p = 0.82); **B** Overall survival according to time to surgery (p = 0.97); **C** Disease free survival according to time to decision (p = 0.56); **D** Overall survival according to time to decision (p = 0.5)

Univariate survival analysis using Cox model showed no significant statistical differences with a 14-day TTS threshold (Fig. 2) for DFS (p = 0.82) or OS (p = 0.97). No difference was shown for DFS either with a 7- (p = 0.22), 28- (p = 0.33), 60- (p = 0.79) or 75- (p = 0.88) day delay. OS and DFS were not different in both groups when comparing extreme delays (Table 3). TTD did not impact either DFS or OS, regardless of the delay cut-off (Table 3).

**Table 3:** Overall and disease-free survival analyses according to different times to decision and time-to-surgery cut-offs

Cut-off	Number of patients		Overall survival			Disease-free survival		
	Lower cut-off	Higher cut-off	p	OR	CI 95%	p	OR	CI 95%
Univariate analysis								
Time to decision								
≤ 7 days vs. > 7 days	18	147	0.59	0.89	0.57–1.38	0.56	0.88	0.58–1.34
≤ 14 days vs. > 14 days	59	106	0.50	0.86	0.55–1.34	0.56	0.88	0.58–1.34
≤ 28 days vs. > 28 days	104	61	0.79	1.07	0.65–1.76	0.68	1.10	0.69–1.8
≤ 60 days vs. > 60 days	151	14	0.78	0.88	0.35–2.19	0.79	1.09	0.6–1.97
≤ 7 days vs. > 28 days	18	61	0.96	0.98	0.58–1.68	0.97	1.01	0.61–1.68
Time to surgery								
≤ 7 days vs. > 7 days	18	147	0.21	1.43	0.82–2.52	0.22	1.4	0.81–2.41
≤ 14 days vs. > 14 days	59	106	0.97	0.99	0.67–1.46	0.82	0.95	0.66–1.39
≤ 28 days vs. > 28 days	104	61	0.38	1.2	0.80–1.77	0.33	1.21	0.83–1.76
≤ 60 days vs. > 60 days	151	14	0.88	0.95	0.52–1.74	0.79	1.09	0.6–1.97
≤ 75 days vs. > 75 days	157	8	0.84	0.93	0.3–1.99	0.88	1.06	0.49–2.28
≤ 7 days vs. > 60 days	18	14	0.34	1.5	0.66–3.41	0.26	1.6	0.71–3.59
Multivariate analysis^a^								
Time to surgery								
≤ 14 days vs. > 14 days	59	104	0.44	1.18	0.76–1.82	0.99	1.00	0.66–1.51

*vs* versus; *OR* odds ratio; *CI 95%* confidence interval 95%

^a^Adjusted analysis for vein resection, jaundice, and hemorrhage

Adjusted OS (on organ and vein invasion, severe complications, and adjuvant chemotherapy) and adjusted DFS (on vein invasion, organ resection, severe complications, R status, N status, and adjuvant chemotherapy) were not significantly improved with a shorter TTS, inferior or equal to 14 days (p = 0.44(Table 3).

Delay had no influence on the occurrence of fistula (p = 0.44), hemorrhage (p = 0.35) and severe post-operative complication (p = 1) (Table 4). Multivariate logistic regression analysis adjusted on significant factors selected from the univariate analyses did not show any statistical significance concerning fistula (p = 0.41), severe post-operative complication (p = 0.82) or hemorrhage (p = 0.59) (Table 4).

**Table 4:** Univariate and multivariate analysis comparing the 90-day postoperative morbidity for a 14-days time to surgery

	≤ 14 days (N = 59)	> 14 days (N = 106)	Univariate analysis	Multivariate analysis	
	n (%)	n (%)	p	p	OR [CI95%]
Hemorrhage (versus absence)^a^	10 (25.6)	11 (16.2)	0.351	0.59	1.34 [0.46–3.94]
Fistula (versus absence)^b^	12 (31.6)	28 (41.2)	0.442	0.41	0.66 [0.24–1.16]
Severe complications (versus Clavien < 3)^c^	15 (25.4)	26 (24.5)	1	0.82	0.75 [0.06–9]

*n* number of patient; *N* total per category

^a^Adjusted for jaundice, superior mesenteric vein invasion, and T status

^b^Adjusted for jaundice, tumor localization and type of resection

^c^Adjusted for body mass index, lymph node invasion, invaded/sampled lymph nodes ratio and superior mesenteric vein invasion

## Discussion

In our study, TTS—considered as the time from the first specialized interview to surgery—had no impact on DFS and OS in patients with upfront resected PA. Hemorrhage, fistula, or severe post-operative complications rate were not different, regardless of TTS. This is the first study to evaluate the impact of TTS on the 90-day morbidity.

Shortening time to treatment has been a promising approach to improve survival. TTS has been set as a quality care index in other malignancies [6, 7, 22, 23]. As an example, HCC radiofrequency ablations warrant an under five-week management delay to avoid any impact on prognosis [6].

In PA, TTS impact remains unclear. Our results are consistent with most of the previous cohorts that showed no significant influence of TTS on survival rate for patients undergoing a curative intent resection for PA [15, 17, 24]. Eshuis et al. concluded that biliary drainage and prolonged TTS do not impair survival rates in a randomized controlled trial [25]. On the contrary, Swords et al. showed a modestly improved OS (1.8 months) and a higher 30 and 90-day post-operative mortality in shorter TTS, but at the cost of including a large number of patients [26]. Subgroup analysis suggested that shorter TTS could improve resecability rate and prognosis in small tumors [15, 26, 27]. Finally, none of these studies analyzed the consequences of shorter TTS on postoperative complications such as hemorrhage, fistulas or Dindo-Clavien classification.

Published studies, including ours, have failed to demonstrate a significant survival benefit with shorter delays before upfront surgery in PA. However, PA arises from pancreatic parenchyma decades before being symptomatic and diagnosed, suggesting slow growth at early stages [21]. There is also evidence to support the rapid growth of PA, in later natural history once diagnosed, with an estimated time to progression from a T1 to a T4 stage of approximately 14 months [28]. Tumor volume growth could thereby be considered as a factor distinct from the disease stage. Marchegiani et al. showed a TTS effect on survival for smaller tumors (T1 and T2) [27]. Shorter TTS could impact resecability and disease-free survival in this specific patient subgroup. In our study, only 28 patients (16.7%) had a T1 or T2 disease stage, making subgroup analyses futile.

Nevertheless, median TTS seems to be increasing year after year due to the incremental PA incidence and the frequent referral to expert centers [22]. As TTS has no or little impact on prognosis, management strategy could be modified to allow sufficient time to confirm diagnosis in ambivalent cases and integrate preoperative chemotherapy and prehabilitation strategy. A 28-day minimum delay between last liver imaging and surgery remains well established to avoid any curative surgery performed on patients with liver metastases [3].

No link was observed for fistula, hemorrhage, or severe post-operative complications when TTS was shortened. Post pancreatectomy hemorrhage is mainly due to anastomotic leaks causing pseudoaneurysms [29]. Also, when performed early, these interventions are less likely to be the subject of technical debate in multidisciplinary surgical meetings and patients may be less well prepared (uncorrected coagulation, nutritional status, or jaundice). Moreover, significantly higher median bilirubin in the shorter delay group can lead to troubled hemostasis, facilitating post-operative hemorrhage [30].

Our study has several limitations. First, patients with macroscopically incomplete resection (R2) or those who finally did not undergo a curative surgery, despite an initially resectable primary tumor, were not included. These patients could potentially have longer delays explaining tumor progression. Unfortunately, they were not considered in our analysis avoiding performing an intention-to-treat analysis. Vein resection rate was higher in the over 14-day delay group. This significative difference could be explained by a higher number of patients with borderline resectable disease in this group. Unfortunately, no systematic preoperative imaging review was performed, which did not allow us to better assess those patients. Furthermore, patients who waited longer without any treatment but finally underwent a curative surgical procedure may have had slower-progressive disease with a better prognosis. Preselecting candidates for curative surgery on their time to progression in the setting of such an aggressive disease seems unethical. Also, 116 patients (69%) were diagnosed upon jaundice presentation, while only 64 (38.1%) experienced a radiological or endoscopic biliary drainage. Patients treated before 2010 were more frequently resected with higher bilirubin levels. Moreover, the power of the study could be insufficient due to the limited number of inclusion centers and retrospectively included patients. Finally, three of the four inclusion centers, including 92.9% of the patients, were tertiary hospitals. We cannot exclude a selection bias and a confusion effect due to highly skilled techniques developed in these centers with low complication rates.

Surgery remains the only existing treatment to cure PA [3]. Recent progress with polychemotherapy regimens and better patient selection for surgery has modestly improved overall survival [31, 32]. Moreover, new management strategies including neoadjuvant and induction chemotherapies are to be interpreted differently from passive delay where no therapeutic interventions occur. New perspectives must be found to increase the survival and quality of life of these patients. PA may not be the most suitable malignancy to study TTS as a quality metric. However, the COVID-19 pandemic has called for urgent case hierarchization and has thereby raised new questions about carcinologic surgical priorities. Ongoing multicentric CAPANCOVID-19 (https://clinicaltrials.gov/ct2/show/NCT04406571) tries to measure the impact of the COVID-19 pandemic causing prolonged management delay, from surgery to palliative situations.

We showed that TTS seems to have no impact on OS, DFS and 90-day postoperative morbidity in patients with resected pancreatic adenocarcinoma. Other trials need to be carried out to understand the role of TTS in smaller tumor sizes.

## Data Availability

The datasets used and/or analysed during the current study are available from the corresponding author on reasonable request.
